# Collaborative Patterns, Productivity, and Research Impact in the Careers of Star Researchers in a Japanese Semiconductor Company

**DOI:** 10.3389/frma.2020.575862

**Published:** 2020-09-29

**Authors:** Akira Maeki, Cristian Mejia, Yuya Kajikawa

**Affiliations:** ^1^Research and Development Division, Hitachi America, Ltd., Santa Clara, CA, United States; ^2^Department of Innovation Science, School of Environment and Society, Tokyo Institute of Technology, Tokyo, Japan; ^3^Institute for Future Initiatives, The University of Tokyo, Tokyo, Japan

**Keywords:** star researcher, creativity, productivity, hot streaks, semiconductor, patents

## Abstract

This study analyzes how characteristics in the careers of star researchers affect the outcomes of research and development (R&D), based on a case study in a Japanese semiconductor company. By analyzing the collaboration network of patent coinventors in the company, we observe that long-term exposition and collaboration with other high-achieving researchers play a significant role in determining a successful career, that is, in terms of productivity and impact. Also, a deeper exploration of the characteristics of a selected group of star researchers in a company's R&D division helped to identify that it takes 10–15 years to generate remarkable achievements in the form of filing patents that are widely cited at a later stage. This period is followed by low productivity, thereby revealing productivity peaks such as those observed in the artistic and scientific careers but at different times. Industry researchers tend to follow a more fixed pattern. Additionally, we analyzed the influence of having star researchers in coinventor teams. Our results suggest that staying aligned in one research direction, long-term exposure to a diverse group of researchers, and early mentorship helped the researchers in our study to attain their achievements.

## Introduction

Understanding the path to major accomplishments in the careers of individuals whose job requires a creative component has been the subject of inquiry for many scholars. Especially, when such creative efforts can be translated into economic and social value, as is the case for companies and their R&D divisions.

Researchers in an industry devote considerable creative effort when generating innovative ideas, usually represented as patented inventions. This creative process is only a recombination of previous resources and ideas. Individual expertise is critical in helping organizations to generate new knowledge and recombine existing ideas to create innovative applications (Glynn, 1996). Hence, to be at the forefront of innovation, companies are keen to understand how productivity changes throughout a researcher's career, that is, whether it follows a predictable pattern and what are the underlying factors that trigger such patterns. Previous research has demonstrated that patterns of outstanding achievements can be observed among artists and academics alike, where productivity peaks can appear at any stage of their individual careers (Sinatra et al., 2016), and their most important accomplishments are clustered together in time (Liu et al., 2018). However, this is yet to be validated for careers in industry research.

The present study fills this gap by addressing the following questions: if researchers in an R&D division show a predictable pattern of productivity in their careers, if highly productive researchers help to nurture other productive researchers through a knowledge spillover effect, and what are the perceived and possible causes of the productivity boost when observed.

**Figure 1:**
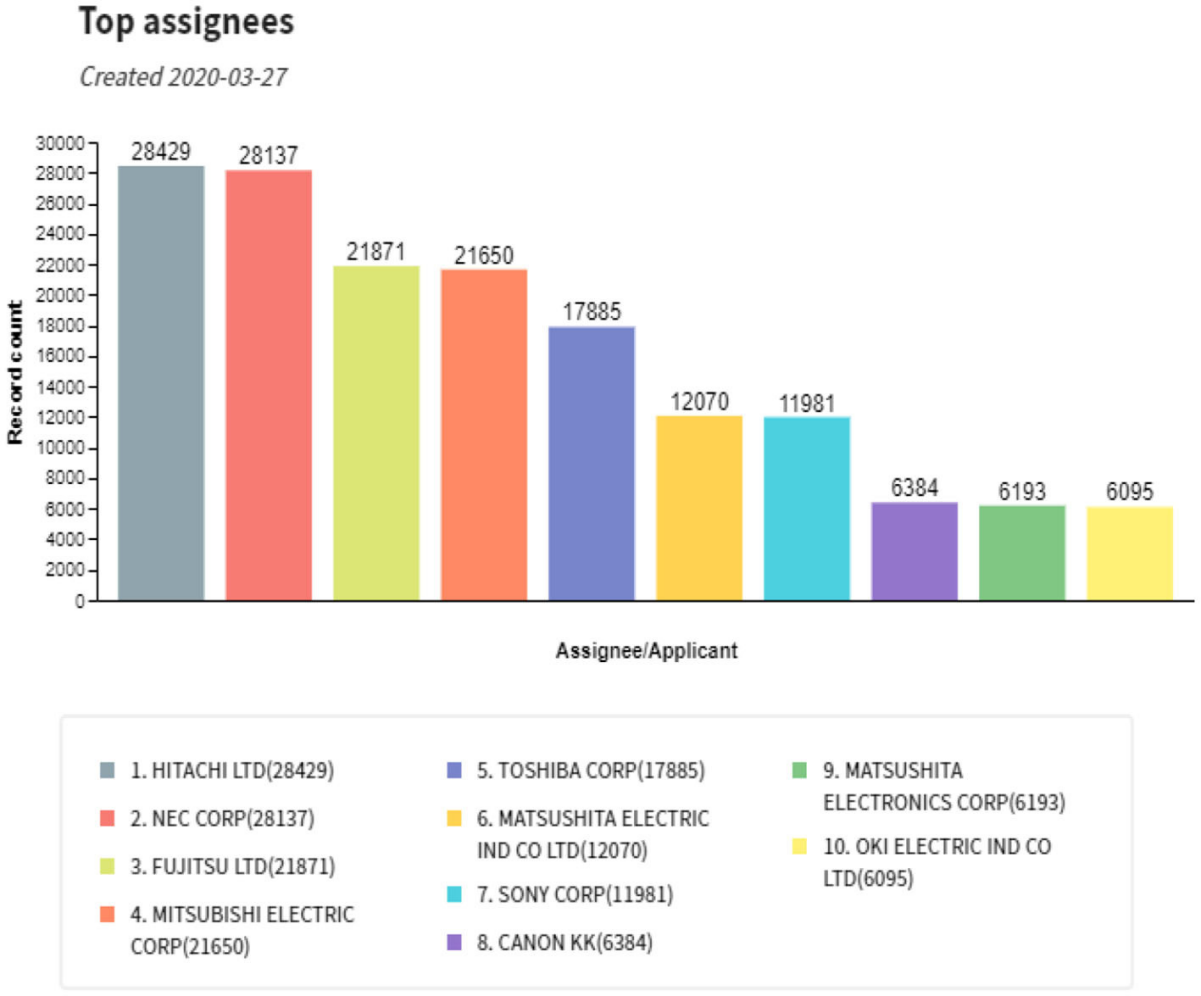
Top international assignees for semiconductor patents during the 1980s and 1990s. Note that all the top ten assignees were Japanese companies.

To answer these questions, we present a case study in a Japanese semiconductor company. First, we analyze the collaboration network of coinventors credited in the company's patents to identify the most productive and impactful inventors. Subsequently, we analyze the interactions of those “star researchers” with their coinventors to infer their key characteristics and differentiators. Second, we narrow the scope down to a specific R&D division within the company. Hence, we aim to investigate characteristics and research careers that affect the research outcomes via quantitative analysis of patent statistics. Researchers in the present study were productive from the 1980s to 1990s when the Japanese semiconductor companies had captured the highest share of the global market and were in the middle of a competitive environment with the pressing expectation of always scaling Moore's law (Moore, 1998). Figure 1 shows the most prolific companies, worldwide, in terms of filing semiconductor patents during the 1980s and 1990s. Even though the figure summarizes international companies, the ten largest were Japanese.

## Previous Literature

### Productivity, Creativity, and Innovation

Creativity is the source of organizational innovation (Amabile, 1982), and a limited asset for new product development. Consequently, abundant literature is dedicated to understanding the factors that influence creativity in individuals, and the organizational aspects that nurture creativity within organizations (Hackman and Oldham, 1975; Ford, 1996; Hunter et al., 2007). For instance, rewards for performance (Amabile et al., 1986; Baer et al., 2003), or a competitive environment (Shalley and Oldham, 1997) are among the many factors that contribute to environments that stimulate creativity. Thus, it is clear that management plays a key role in promoting and sustaining practices that allow researchers to achieve their full potential (Oldham and Cummings, 1996).

An environment that stimulates the creativity of members of the organization is the starting point for the consecution of output that may bring value to the company. The performance of innovators is observed by the number and quality of outputs (e.g., patents). In the literature, output quality is assessed as the impact creators bring to the community of peers, society, or organization, commonly represented by highly-cited patents or papers, breakthroughs, economic value, or awards. The combination of productivity and impact together constitute the achievements of the creators.

In the context of new product development, prior literature discusses that, overall, there are less impactful outputs on the business in relation to R&D spending (Cooper and Edgett, 2008). Therefore, frameworks have been developed to smooth the process between individual creativity and organizational innovation that maximize the value from research to development and commercialization (Griffin et al., 2009). Generally, the alleged frameworks highlight the team as the key element of creativity.

The process of knowledge creation and recombination occurs at the individual level (Glynn, 1996; Crossan et al., 1999). However, when knowledge grows exponentially, it becomes a burden for the individual researcher to gauge the breadth of expertise necessary for the recombination to occur (Jones, 2008). Hence, organizations leverage creative capacities by organizing teams of experts in various fields (Rulke and Galaskiewicz, 2000; Tiwana and McLean, 2005). This exposes researchers to multidisciplinary knowledge from where they can draw on new ideas (Hargadon and Sutton, 1997), ultimately using the team as a catalyst for creativity.

In terms of researchers' accomplishments, it has been demonstrated that having access to an impressive breadth of knowledge leads to higher productivity (Burt, 2004; Fleming et al., 2007). Meanwhile, researchers' breadth and depth of expertise have a higher impact on innovation (Boh et al., 2014). This does not necessarily mean there is a tradeoff between productivity and impact of creative activities, both are attainable (Shalley, 1991). However, such mastery is observed only among a few individuals within the organization (Boh et al., 2014).

### Star Researchers and Hot Streaks in Their Creative Careers

Individuals who attained remarkable achievements, which impacted their organizations or scientific communities, are identified as “star scientists” (Han and Niosi, 2016), “hero scientists” (Griffin et al., 2009), “exemplary creators” (Gardner, 2011), and “hyper prolific authors” (Ioannidis et al., 2018), each of them with nuanced definitions. Most literature on “star scientists” focuses on the biotechnology domain (Han and Niosi, 2016) to understand how these scientists participate in the process of technology diffusion, beyond academia, through University spin-offs (Zucker et al., 1998; Zucker and Darby, 2007). The study of temporal career patterns for characterizing emergence and timing of outstanding achievements, specifically for new product development, is still lacking.

Although some individuals are productive and create impactful inventions, this is not constant during their research life (Mumford, 1984). Earlier studies on patterns of creativity in the career of individuals can be traced back to 1835 to the work of Quetelet and the application of historiometry (Quetelet et al., 2013). However, the relationship between creativity and age was formally established by Lehman (1953) in his seminal work on age and achievement. He investigated how researchers' best results tend to peak during their early thirties and decrease in their forties. Similar results were observed in later studies (Pelz and Andrews, 1966; Dalton and Thompson, 1971). Simontons (1977, 1988, 2000) research on career trajectories and landmarks clarified the causalities of such peaks of creativity, either in the form of outstanding productivity or remarkable impact. Although first demonstrated in the careers of artists, he observed a pattern of remarkable achievements as a function of the individual's age. This curve takes the form of a bell and is arguably a combination of the ideation rate, which decreases as we get old, and elaboration rate, which improves with age. The former is higher at the early stage of the creative career as it is easy to establish linkages in a theme that is new when thoughts are still unorganized. With experience, the frame of thinking becomes fixed making it more difficult to accept new ideas. However, cognitive elaboration rises, thus helping to distinguish good ideas from bad ones.

Remarkable achievements have also been studied in athletes, musicians, and chess players, where it is found that top professionals in these areas reach a plateau after 10,000 h of training (Ericsson et al., 1993). In a more comprehensive study that focuses on the careers of top scientists, artists, and filmmakers, evidence shows the existence of hot streaks (i.e., periods of great productivity), however, independent of time appearing at any point of their careers (Liu et al., 2018).

Regarding new product development, as illustrated by the studies of Boh et al. (2014), who present a case study of 3M, enterprise researchers are categorized into three groups: specialists, generalists, and polymaths, based on the breadth or depth of expertise. They discuss how each group contributes toward generating economic value to the company in the form of converting innovations into products. Nishimura et al. (2000) studied the perceived factors that led researchers in a pharmaceutical industry in Japan to generate more research outputs for their organizations, dividing the subjects into two generations and evaluating aspects other than age. They found that the performance indicator for product development, which measures patent activity, was rather stable in the two groups. Beyond the above-mentioned criteria, there is no sign of other literature studying creative performance in the careers of industry researchers for new product development.

There are, however, other settings where the presence of star scientists has been the subject of examination. Research shows that some industries benefit more from a sole “super” individual than a team; however, when such a “star researcher” is not available, then it creates a context when a “fantastic” team must be configured (Taylor and Greve, 2006). The value of star researchers is that they are not only knowledge producers but also collaborators enhancing the capabilities of those around them through within-firm interactions (Grigoriou and Rothaermel, 2014), thereby, generating a spillover effect of their expertise within the organization and beyond (Zucker et al., 2002).

The concept of spillover is commonly associated with knowledge and economic transfer from one region to another, or across industries. Knowledge flows can also be observed at more localized levels, within an industry or within a company, for instance when skills are transferred from experts to a novice (Bjursell and Florin Sädbom, 2018). In the context of innovation, collaborative patterns in patent activities have been faster in information diffusion compared to weak ties (Wang et al., 2017). Furthermore, companies with large in-house capabilities benefit more by internal and self-promoted collaboration, than by collaborations due to spillovers from the outside or motivated by external factors (Grillitsch and Nilsson, 2015). In a parallel setting, collaboration among researchers from different academic institutions has been found to be valuable in enhancing research quality in terms of the number of high-impact articles published by the institutions involved in such collaborations. The benefits of collaborations goes beyond the individuals and institutions, also reaching benefits at the country level (Aldieri et al., 2018). Overall, there is a paucity in the literature of studies tackling knowledge spillover from highly achieved researchers to other researchers within a single company.

The present study explores the career of star researchers in the context of their creative achievements, represented as patents and awards in new product development; and how these researchers benefit themselves and others by observed collaborative patterns. The remainder of the paper contains the definitions, methodological approach, and results. Finally, implications of the productivity peak, the role of star researchers as part of the creative network of inventors in the company, and the late stages of career are discussed.

## Data and Methods

**Figure 2:**
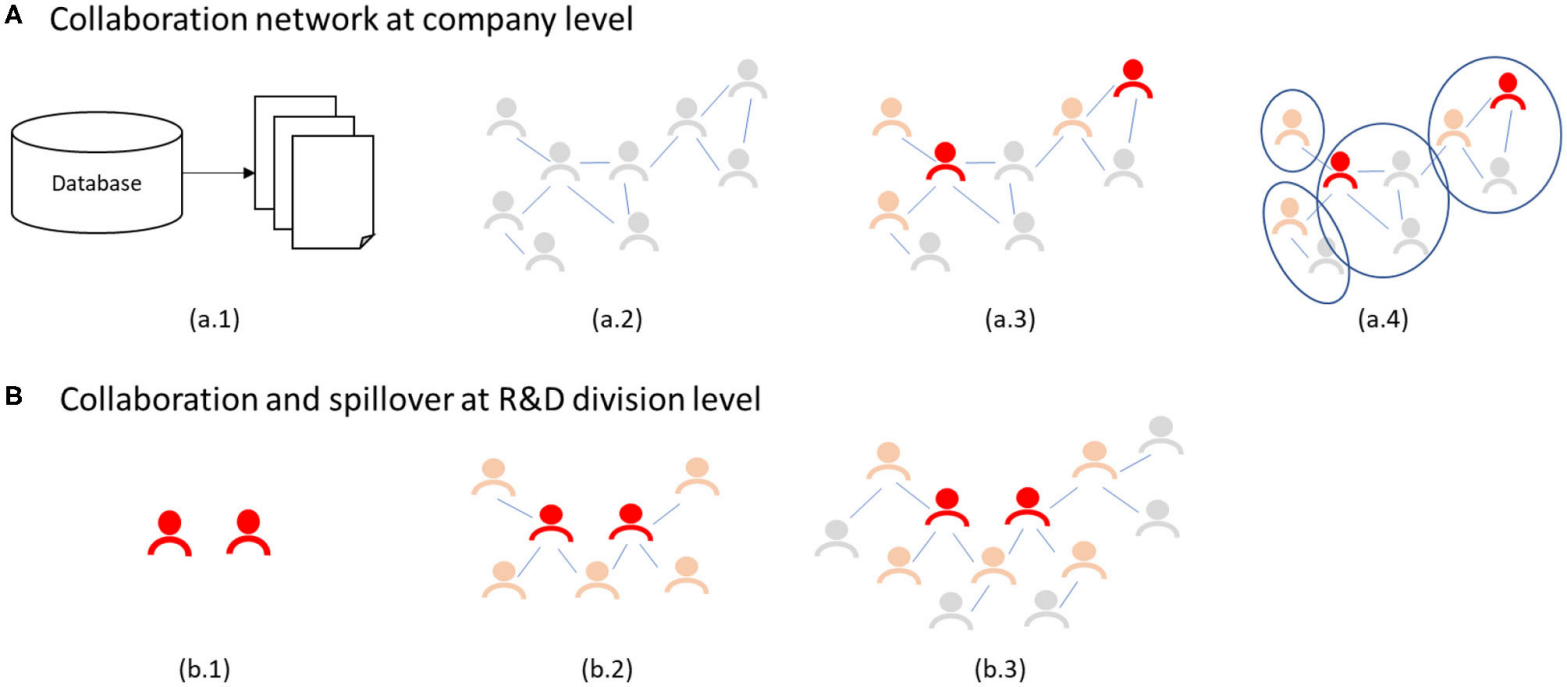
The methodological approach in this research. At the company level patents are extracted from a patent database **(a.1)**; a network of inventors is created where 2 inventors are connected if they co-invented a patent **(a.2)**; start researchers and other highly achieved researchers are identified and their characteristics studied with binomial logistic regression **(a.3)**; clusters of inventors are obtained and analyzed. At the R&D level, a subset of star researchers that worked in the same division was identified **(b.1)**; direct collaborators and second level collaborators were identified **(b.2, b.3)**; and their career characteristics were studied.

The present study is divided into two parts. First, it is an overall assessment of the collaboration network of individuals in a company. Second, it presents a detailed analysis of star researchers at the R&D level. Figure 2 summarizes the methodological approach for both parts.

To study the collaboration network, we analyzed the company's patents related to semiconductors that were filed in Japan. Data were collected from Derwent Innovation, a patent data aggregator that collects data on granted patents and applications for more than 40 issuing authorities, including Japan. Derwent Innovation is a database commonly used in patent analysis because of the added benefit of English translations of titles and abstracts, besides the original language of the patent. Moreover, inventor names are standardized, and this helps in reducing the possibility of wrongly assigned patents to inventors with the same names. To collect the data, we applied the following query: “ALLD=(“SEMICONDUCTOR^*^”) AND CO=(“^*******^”) AND AD>=(19800101)^1^.” With this, we pulled patents with keywords “semiconductor” or “semiconductors” in any of the text fields, considering applications filed since the 1980s. We retained those granted and filed in Japan only. Data were retrieved on March 27, 2020.

For each patent, we examined the inventor field, where the standardized names of inventors for each patent is listed. We implemented cleaning steps such as uppercasing the names and removing symbols or extra whitespaces that could affect the correct attributions of inventors. Once cleaned, we built a network of coinventors. Each inventor is treated as a node, and two inventors get connected if they co-invented a patent. The more a pair of inventors work together and are successfully granted a patent, the stronger their connection in the network. To avoid incidental inventors, we retained inventors with five or more patents, as those with modest contributions may refer to technicians, interns, or other individuals who may have contributed to a patent occasionally but were not employed by the company, or who worked for the company for a short period.

Subsequently, we proceeded to identify the star researchers. We define star researchers as individuals satisfying the following two conditions:

1. Productivity: Top 1% productive individuals by the number of patents.
2. Impact: Individuals who contributed to two or more of the top 1% cited patents.

The selected threshold was challenging for any research to achieve. Specifically, star researchers appear as inventors in at least 72 patents and have at least 2 patents with 74 citations or more. We focus on inventors with two or more highly cited patents to avoid one-time collaborators who may have participated in the invention of a highly cited patent. Inventors were further classified as follows: “non-star researchers A1” (NSR-A1), or those who accomplish either condition 1 or 2 and “non-star researchers A2” (NSR-A2) comprise the remainder.

We then fitted two binomial logistic regression models to identify variables that have maximum influence in labeling an individual like a star researcher for this company. One model compared the extreme case of star researchers against NSR-A2, while the other compared the star researchers vs. both NSR-A1 and NSR-A2. Due to the long tail of NSR-A2 researchers with low patent productivity, we focus this part of the study on those with 30 or more patents during their careers in the company.

We considered a star researcher as a dependent variable (which is binary, a researcher is either a star researcher or not) and compared it to six independent variables:

- Active years: number of years from the first to the last patent
- Median team size: average number of coinventors
- Max team size: The largest number of coinventors for a single patent
- Number of star researchers' coinventors: total number of star researchers' coinventors during his/her research life
- Number of NSR-A1's co-inventors: total number of NSR-A1's coinventors during his/her research life
- Number of NSR-A2's co-inventors: total number of NSR-A2's coinventors during his/her research life.

Besides the comparison of characteristics of star researchers and the rest of researchers, in the network, we could also identify groups of inventors who worked together frequently. These clusters of inventors were derived from the network using the Louvain algorithm (Blondel et al., 2008). The clustering analysis helps to reveal career-long patents of collaboration among the star researchers in the company.

Second, we studied the lifetime productivity of a subset of star researchers in a specific R&D division in the company. We focused on this subset because we were granted access to the career particulars of researchers in this division, including information on their whereabouts following retirement. We could therefore keep track of any changes in their interest areas. This is relevant because the company spun out its semiconductor division as a new subsidiary; some researchers remained in the original company but changed their expertise, while others were transferred to the new company and continued working on their projects. Others moved to work as a professor in a University expanding their professional networks. To narrow down the star researchers in this R&D division, we considered those whose achievements have been recognized as impactful in a social context, that is, their achievements have been publicly recognized by peers in specialized societies with a prestigious award, or they are recognized as leaders by senior experts on the field. They are researchers awarded with the Yamazaki–Teiichi prize^2^, which is a major industrial award in Japan, they have either been distinguished with the IEEE Fellow grade^3^ or referenced by two anonymous senior executives.

Furthermore, we analyzed the knowledge spillover effect of this subset of highly productive researchers to other researchers by assessing their patent activities as coinventors. We consider spillovers when coinventors become highly productive in a posterior time if previously they had co-invented a patent with a star researcher. To create the association between star researchers and others, a list of coinventors was created for each star researcher. Subsequently, for each star researcher, we selected three non-star researchers by a random selection as “non-star researchers B1” (NSR-B1). We replicated the process to randomly select a second degree of coinventors (i.e., coinventors of coinventors) as “non-star researchers B2” (NSR-B2). The performance of these groups was compared over time to assess the spillover effects. To supplement the above patent analysis, we conducted five interviews, where two of the five interviewees are star researchers, and the rest are department managers from the same company. The results of the interviews are shown in the Appendix (Supplementary Material).

## Results

### Overall Assessment of the Collaboration Network in a Company

Since 1980, the company has been granted 44,636 semiconductor-related patents filed in Japan and attributed them to a total of 18,843 different inventors. About 38.1% of them participated in the invention of just a single patent. To avoid inventors with limited patent activity, we analyzed the subset of 6,057 inventors having five or more patents during that period. These inventors were further classified into three categories, as shown in Table 1.

**Table 1:** Classification of study individuals.

**Category**	**Number of inventors**
Star researchers	139
NSR-A1	535
NSR-A2	5,383

To understand the differences between star researchers and the rest of researchers, we fitted a binomial logistic regression model considering the career-long characteristics of both type of researchers, including the number of active years filing for patents, median, and max team size when they worked as coinventors, and the number of other star researchers, NSR-A1, and NSR-A2 collaborators during their careers as semiconductor-related inventors in the company. These served as independent variables, mainly to infer if an individual is labeled as a star researcher or not. Table 2 shows the results for the models.

**Table 2:** Dependent variables of a binomial logistic regression explaining star researchers vs. NSR-A2 (above), and star researchers vs. all other researchers (below).

**Estimate**	**Estimate**	**Std. Error**	***Z*-value**	**Pr(>|z|)**	**Significance**
(Intercept)	−6.6189	0.9614	−6.8846	6.E-12	***
Active years	0.0429	0.0314	1.3679	2.E-01	.
Median team size	−0.4507	0.1869	−2.4116	2.E-02	*
Max team size	0.0743	0.0565	1.3147	2.E-01	
The number of NSR-A2's coinventors	0.0717	0.0130	5.5228	3.E-08	***
The number of NSR-A1's coinventors	0.1701	0.0377	4.5174	6.E-06	***
The number of star researchers' coinventors	0.2332	0.0499	4.6776	3.E-06	***
(Intercept)	−4.7323	0.6942	−6.8166	9.E-12	***
Active years	0.0191	0.0225	0.8496	4.E-01	
Median team size	−0.3907	0.1337	−2.9218	3.E-03	**
Max team size	−0.0383	0.0304	−1.2593	2.E-01	
The number of NSR-A2's coinventors	0.0574	0.0077	7.4879	7.E-14	***
The number of NSR-A1's coinventors	0.0521	0.0207	2.5175	1.E-02	*
The number of star researchers' coinventors	0.2275	0.0281	8.0905	6.E-16	***

Significance levels: ***P < 0.001; **P < 0.01; *P < 0.05; and P < 0.1.

The first comparison is between star researchers and NSR-A2, in this model we did not compare star researchers to NSR-A1 as our intention is to understand the characteristics of those with highly remarkable achievements vs. those who do not have them. In fact, NSR-A1 can be said to have remarkable achievements and maybe on their way to becoming star researchers themselves. We found that collaborations with other researchers during their careers were the most influential explanatory variables in the model. This suggested that the more they interacted with a variety of inventors during their career, the more likely they were to achieve the characteristics of star researchers as defined in this study. The number of active years as inventors—from their first to their last patent—played a less important role. The median size of the team had a negative coefficient, suggesting that smaller teams were more significant in defining a star researcher, although the team size had less influence compared to long term exposure to a plurality of other researchers. This was also confirmed when observing the max team size the researchers had participated in, which showed no significance in the model. For a star researcher, participation in larger sized teams had no influence.

The second model compared star researchers with the rest of researchers (i.e., both NSR-A1 and NSR-A2) showing similar results. Long-term exposure and collaboration with a plurality of other researchers had a positive and significant influence, although comparatively less significant for NSR-A1. This implied that teams consisting of star researchers and a novice would be beneficial to the inventors' careers. The number of active years was not significant in the model, while, again, the median team size had a negative coefficient, this time with a significance at *P* < 0.01. Under this model, it is more likely to be recognized as a star researcher when the researcher had participated in teams of a relative smaller size. Participation in bigger teams had no influence on the career of star researchers.

**Figure 3:**
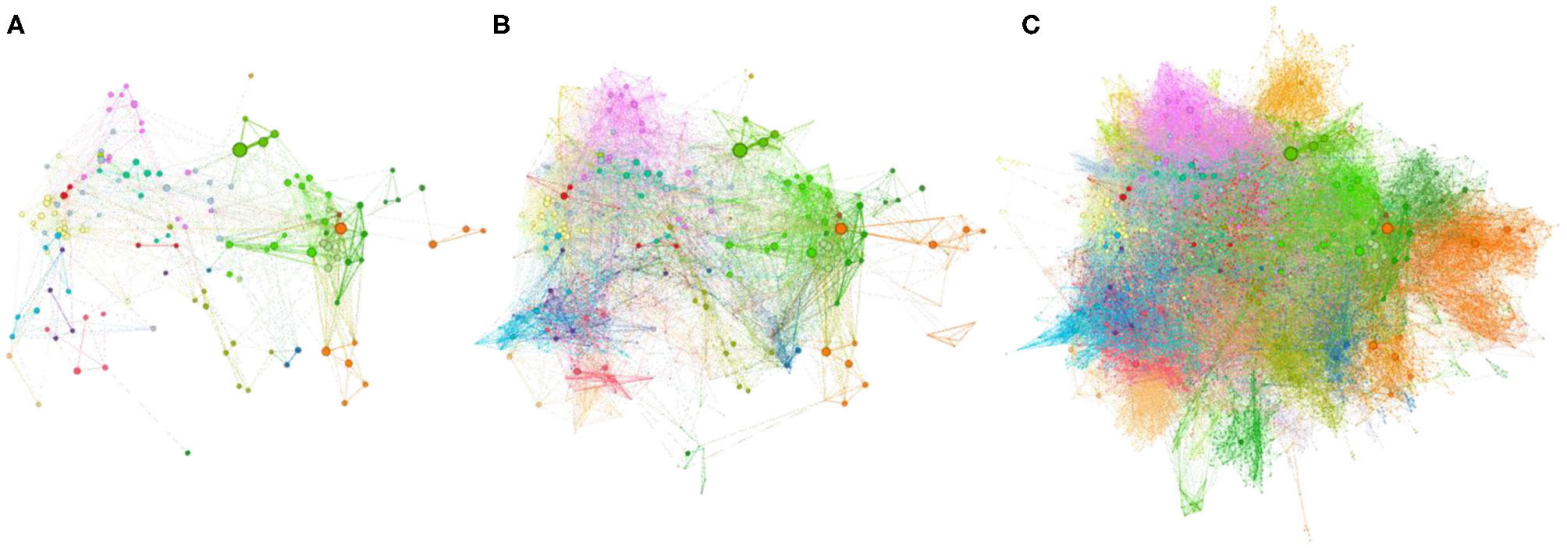
Collaborative networks of inventors in semiconductor-related patents from 1980 to present. Clusters of frequently co-occurring coinventors are represented in different colors. The size of the node is relative to the number of patents per inventor. From left to right: **(A)** star researchers; **(B)** star researchers and NSR-A1; **(C)** all researchers in the study.

The collaboration network of these individuals is shown in Figure 3. We found 34 clusters or groups of inventors who coinvent together. The network is cohesive, with all the individuals allocated to a single component. Figure 3A shows the network filtered to indicate the location of star researchers. Despite eliminating most of their collaborators in the network, star researchers remain as a single component, meaning that they do not exist in isolation and have collaborated at some point with other star researchers, regardless of the others belong to the same cluster. Table 3 summarizes the clusters.

**Table 3:** Summary of clusters of inventors: a. Cluster number, b. Inventors, c. Star researchers, d. NSR-A1, e. NSR-A2, f. Patents, g. Average Application Year, h. Average Citations, i. Top IPC subclasses.

**a**.	**b**.	**c**.	**d**.	**e**.	**f**.	**g**.	**h**.	**i**.
1	579	14	87	478	5259	1992.7	8.6	H01L:3983; G11C:1448; H03K:664
2	439	13	39	387	4372	1991.3	6.1	H01L:4134; H05K:326; B29C:175
3	407	6	51	350	3057	1997.0	7.8	H01L:2401; H01J:852; G03F:738
4	371	4	15	352	3221	2003.8	4.4	H01L:2450; C09J:848; C08G:565
5	351	5	37	309	3749	1991.8	5.1	H01L:2647; H01S:1220; G02B:218
6	334	8	23	303	3173	1989.9	6.4	H01L:2881; G11C:338; G01N:115
7	296	9	14	273	2395	1993.2	5.6	H01L:1937; H02M:345; H03K:131
8	281	1	3	277	2294	2003.6	3.5	H01L:2189; C23C:995; B65G:79
9	258	16	37	205	3320	1992.8	7.4	H01L:3154; G03F:225; C23C:182
10	254	3	21	230	2127	1996.4	6.8	H01L:1711; H05K:270; G01L:120
11	233	8	34	191	2134	1993.4	9.1	H01L:1815; H05K:326; G01R:189
12	221	5	4	212	1660	2000.9	3.9	H01L:1287; C08L:449; C08G:412
13	217	0	15	202	1549	1995.4	6.6	H01L:1154; G02F:251; C23C:96
14	204	4	13	187	2158	1989.2	4.7	H01L:1956; H03K:129; G11C:70
15	193	2	29	162	1565	1997.7	5.7	H01L:1468; H05H:349; C23F:267
16	192	5	4	183	1793	1996.3	3.7	H01L:1447; H05K:168; H01B:110
17	191	1	2	188	1230	1998.5	6.1	H01L:585; G11B:204; H05K:125
18	178	5	37	136	1649	1993.4	9.1	H01L:1413; G01N:463; G03F:261
19	144	18	32	94	2225	1991.4	9.4	H01L:2086; G11C:165; C23C:64
20	142	1	4	137	1114	1998.2	5.5	H01L:803; H05K:193; C22C:90
21	141	1	8	132	1706	1998.7	2.9	H01L:1404; C30B:486; C23C:160
22	93	0	1	92	597	1994.2	4.4	H01L:397; G01T:63; H01J:63
23	78	0	0	78	616	1994.1	4.9	H01L:442; B23K:70; H04N:65
24	72	0	3	69	597	1999.8	7.8	H01L:498; H05K:46; B01D:45
25	44	5	4	35	533	1991.1	6.4	H01L:434; C08G:195; C08L:132
26	40	4	8	28	868	1992.3	4.5	H01L:784; H05K:97; C09J:40
27	37	1	4	32	331	1992.3	11.5	H01L:303; G11C:73; H03K:17
28	17	0	0	17	92	2001.4	2.7	H01L:58; F21S:29; F21V:27
29	13	0	0	13	47	2003.3	1.9	F24F:42; B01D:22; F04D:5
30	13	0	5	8	269	1993.1	8.9	H01L:182; G11C:160; H03K:61
31	8	0	0	8	10	2007.4	3.1	G06F:8; B25C:2
32	7	0	0	7	20	1998.4	12	F04D:11; B05C:7; G03F:3
33	7	0	0	7	22	2015.1	1.3	H01L:21; B22F:7; H02N:6
34	2	0	0	2	6	2003.0	2.3	H04B:4; H01P:2; H03K:2

Clusters are sorted from those having the largest number of inventors followed by how they are distributed as star researchers, NSR-A1, and NSR-A2. Next, we show the statistics of the aggregated patents where these inventors have contributed, including the number of patents, the average year of publication, and average citations. Finally, we obtained the three most common sub-classes of the International Patent Classification (IPC) assigned to the inventor's patents. It serves as a topical signal of interest and expertise to inventors. For most of the clusters, the main IPC subclass is H01L, which expectedly corresponds to semiconductor devices.

In 23 of the clusters, at least one star researcher is present. Clusters where no star researchers are present are smaller in size, except for cluster 13, which groups 217 inventors focusing on optical devices. We did not find strong correlations between the number of star researchers and the remaining variables in the table. However, the presence of a star researcher signals more patent productivity and the inverse of an average number of applications in a year. That is, as observed, star researchers tend to be allocated among groups of inventors who were active and productive in their older years.

### Characteristics of Star Researchers in an R&D Division

#### Career of Star Researchers

Table 4 lists a subset of 15 star researchers that worked in the same R&D division and who achieved special recognition by their community of peers. We complement the table with the year of joining the company and the recognized field of accomplishment, as indicated by the senior executives. Papers of the star researchers reveal that all the researchers graduated from major Universities in Japan, and they provided remarkable research outputs and received promotions. Some have retired but continue to be active as University professors, while a few remain in the company. We surveyed their patenting activities, including the number of patents where the star researchers are either the main inventors or co-inventors and the number of citations received.

**Table 4:** List of star researchers in the R&D department of a Japanese semiconductor company.

**Researcher**	**Year of joining the firm**	**IEEE Fellowship**	**Yamazaki–Teiichi prize**	**Expert acknowledgment**	**Field of major creative achievement**
A	1963	x		x	Two intersection bit cell DRAM
B	1969			x	High speed CMOS-SRAM
C	1969	x		x	Trench capacitor DRAM
D	1974	x		x	Stacked capacitor DRAM
E	1975	x		x	Lifetime of hot carrier
F	1978	x		x	Microprocessor
G	1979	x	x		Low leakage CMOS circuitry
H	1980	x			DRAM stacked memory cell
I	1980	x	x		Low leakage CMOS circuitry
J	1980			x	Microprocessor
K	1980			x	Microprocessor
L	1984	x			Single electron memory
M	1985	x	x		Low leakage CMOS circuitry
N	1985	x			Low power SRAM and MPU
O	1986	x	x	x	3D transistor (Fin-FET)

Table 5 lists the statistics of patent submissions made by star researchers. The table shows the total number of patents where the researchers appear as inventors, followed by the number of patents as primary inventors, and the number of patents as primary inventors with ten or more citations. We computed the active inventive period as the range of years from their first to the last patent submission. Followed by the inventive period as primary inventor considering the range of years between their first and last patent as primary inventors. Then, we computed average productivity per year on primary inventions and highly-cited patents. The overall average for this set of star researchers is 2.08 patents per year.

**Table 5:** Patent productivity of selected star researchers in the R&D department of a company: a. Researcher, b. Total number of patents, c. Number of patents filed as a primary inventor, d. Number of highly cited patents^*^ filed as a primary inventor, e. Period of filing patents (first patent, last patent, period of filing patent/years), f. Period of filing patents as a primary inventor (first patent, last patent, period of filing patent/years), g. Average primary patent per year: (c/f), h. Average highly cited patents^*^ per year: (d/f), i. Proportion of highly cited patents^*^: (d/c).

**a**.	**b**.	**c**.	**d**.	**e**.			**f**.			**g.: c./f**.	**h.: d./f**.	**i.: d./c**.
A	462	104	6	1973	2013	41	1973	2011	39	2.67	0.15	5.8%
B	187	19	1	1972	1991	20	1972	1985	14	1.36	0.07	5.3%
C	178	68	6	1973	2008	36	1973	1997	25	2.72	0.24	8.8%
D	129	74	6	1975	2017	43	1975	2016	42	1.76	0.14	8.1%
E	153	12	1	1976	1996	21	1976	1987	12	1.00	0.08	8.3%
F	118	26	0	1981	2006	26	1981	1993	13	2.00	0.00	0.0%
G	235	70	10	1981	2012	32	1983	2004	22	3.18	0.45	14.3%
H	224	49	4	1982	2010	29	1982	2008	27	1.81	0.15	8.2%
I	199	42	7	1982	2004	23	1982	1998	17	2.47	0.41	16.7%
J	65	34	2	1981	2008	28	1981	2008	28	1.21	0.07	5.9%
K	123	34	1	1981	2003	23	1981	1996	16	2.13	0.06	2.9%
L	144	21	2	1985	2017	33	1985	1993	9	2.33	0.22	9.5%
M	205	88	8	1986	2013	28	1987	2013	27	3.26	0.30	9.1%
N	204	35	5	1985	2015	31	1986	2015	30	1.17	0.17	14.3%
O	172	60	5	1987	2015	29	1987	2015	29	2.07	0.17	8.3%

* *Highly cited indicates receiving ten or more citations*.

Table 5 also shows patents filed only in Japan. These highly productive researchers excelled during the period from the 1980s to the 1990s, which is considered the golden age of the Japanese semiconductor industry, where Japanese companies were highly competitive and enjoyed a large share of the market. Researcher-A was one of the most influential researchers in the laboratory, with several primary inventions and co-inventions to credit. Researcher-F did not file any highly-cited patents but was selected based on reputation. Researchers-G, -I, -N filed a highly cited one for every six or seven patents.

**Figure 4:**
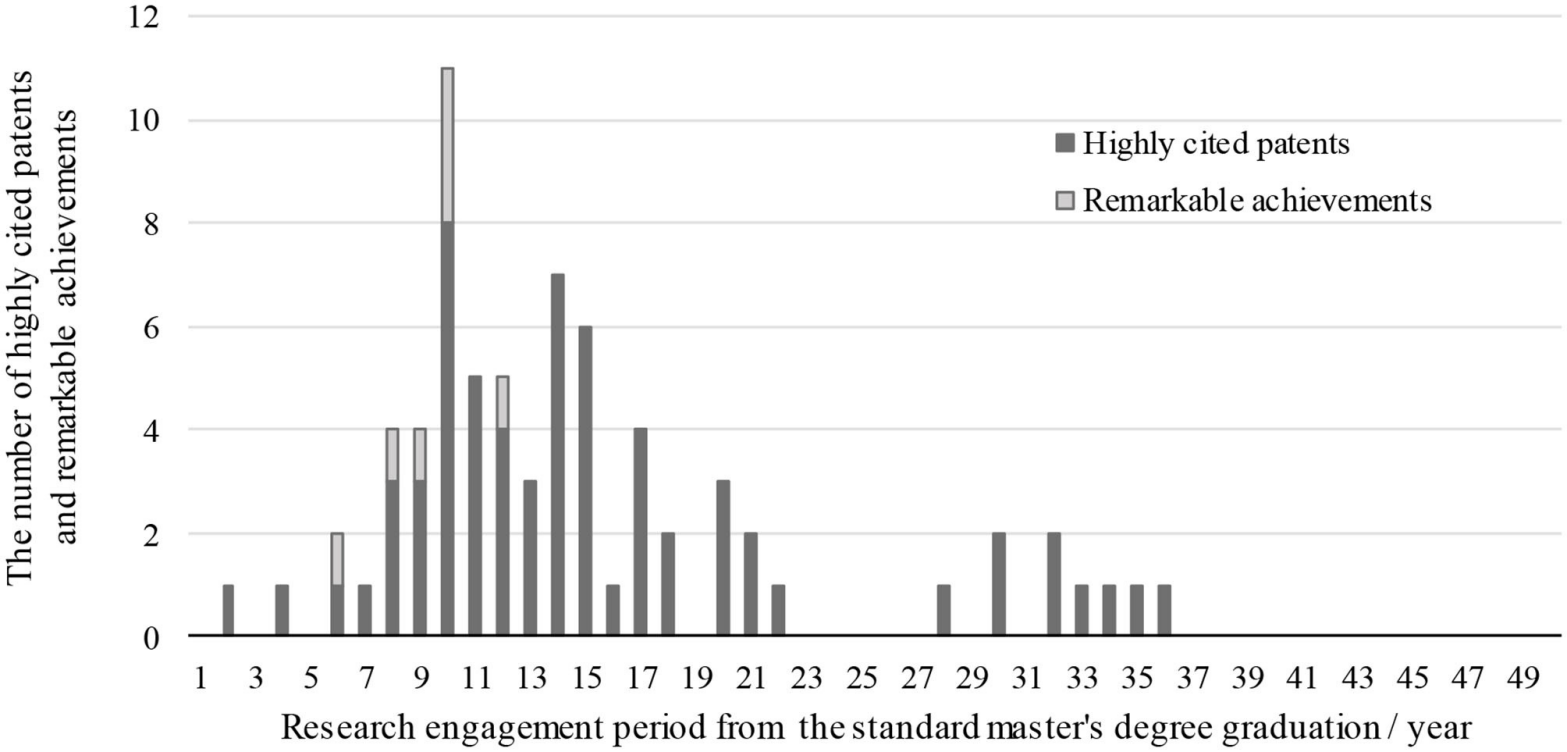
Achievements of star researchers.

Figure 4 shows the number of highly-cited patents, remarkable achievements, and the period of research engagement. The research engagement period was calculated from the year researchers received their master's degree. We considered that year as the starting point of their research activity, aiming to standardize the different paths observed in their careers, as some of them joined the company after they earned a bachelor's degree, while others after completing their doctorate program. Hence, this period reflects the number of years of dealing with research activities from their master's degree to the year of patent submission or remarkable achievement. As shown in Figure 4, highly-cited patents and remarkable achievements were produced 10–15 years after their research career had started. This is proceeded and followed by periods of low activity, revealing a productivity peak in their career. However, even during periods of low productivity, some researchers attained some achievements. To further investigate the variations, we qualitatively investigated those cases. Researchers who graduated from well-known semiconductor laboratories at the graduate University and continue the same research topics after being hired by the company scored remarkable achievements at the early stage of the peak of productivity. This reveals that specialty in the field of semiconductors is a key element for early or continued achievements.

After a period of high productivity, it can be hypothesized that because of their excellence these researchers were promoted to managerial positions, which could act in detriment of the observed patent submissions. To verify this, we checked their patents as primary inventors. 14 out of 15 researchers submitted patents as primary inventors for more than 10 years, 11 researchers did so for more than 16 years, and 9 researchers for more than 20 years. However, for those creating patents after 20 years, their late patents were not highly cited. Additionally, it is worth noticing that even though industry scientists were labeled as “researchers” by the company's conventions, some were not necessarily exempted from performing a heavy load of management activities instead.

#### Spillover Effects

We analyzed the existence of spillover effects from star researchers to other researchers in the same division. Table 6 shows the results. The engagement period was 23.3 years for star researchers, 16.5 years for NSR-B1, and 17.9 years for NSR-B2, on average. In terms of the average number of patents as primary inventors for star researchers, NSR-B1, and NSR-B2 was 2.08, 1.18, and 1.16, respectively. Moreover, the highly-cited patents per year showed a large difference for star researchers, NSR-B1, and NSR-B2 as 0.18, 0.07, and 0.06, respectively. The ratio of highly-cited patents for all patents was 8.4, 5.8, and 4.6%, respectively. Star researchers clearly outperformed the other set of inventors. If spillover effects exist in this department, we could have expected that NSR-B1 would have higher scores than NSR-B2. However, we could not find a large difference, except for the ratio of highly-cited patents between these two: 5.8% for NSR- B1 and 4.6% for NSR- B2.

**Table 6:** Comparison between star researchers and non-star researchers in the R&D department of a company: a. Researcher, b. Statistics, c. Total number of patents, d. Number of patents filed as a primary inventor, e. Number of highly cited patents^*^ filed as a primary inventor, f. Period of filing patents (first patent, last patent, period of filing patent/years), g. Period of filing patents as a primary inventor (first patent, last patent, period of filing patent/years), h. Primary patents per year: (d/g), i. Highly cited^*^ primary patents per year: (e/g), j. Proportion of highly cited patents: (e/d).

**a**.	**b**.	**c**.	**d**.	**e**.	**f**.			**g**.			**h.: d./g**.	**i.: e./g**.	**j.: e./d**.
Star researchers	Average	186.5	49.1	4.3	1980.0	2008.5	29.5	1980.3	2002.6	23.3	2.08	0.18	8.4%
	Maximum	462	104	10	1987	2017	43	1987	2016	42	3.26	0.45	16.7%
	Minimum	65	12	0	1972	1991	20	1972	1985	9	1.00	0.00	0.0%
	Standard deviation	85.8	26.3	2.9	4.8	7.3	6.5	5.0	10.2	9.5	0.68	0.12	0.04
NSR-B1	Average	72.4	19.9	1.2	1982.0	2003.1	22.0	1982.9	1998.5	16.5	1.18	0.07	5.8%
	Maximum	194	72	6	1993	2016	37	1994	2015	36	2.17	0.38	33.3%
	Minimum	12	2	0	1973	1988	10	1973	1983	3	0.40	0.00	0.0%
	Standard deviation	52.6	15.6	1.5	6.0	7.6	7.8	6.1	8.2	8.9	0.49	0.10	0.08
NSR-B2	Average	73.6	21.0	0.9	1985.0	2005.2	21.2	1985.4	2002.3	17.9	1.16	0.06	4.6%
	Maximum	167	72	8	1995	2016	36	1995	2016	36	2.48	0.38	20.0%
	Minimum	11	4	0	1975	1989	4	1976	1987	4	0.52	0.00	0.0%
	Standard deviation	40.9	13.8	1.5	5.0	7.6	7.2	4.8	7.8	7.8	0.45	0.08	0.06

* *Highly cited indicates receiving 10 or more citations*.

## Discussion

Our results indicate the importance of collaboration between inventors across the company. The collaborative network of inventors who co-invented patents since the 1980s in the company shows that star researchers are linked among them and, more importantly, collaborating with multiple other star researchers is a definitory factor in the career of such inventors. This has managerial implications, that is, nurturing high performing industry researchers is necessary to support collaboration with other previously identified stars.

Through focused analysis of the selected star researchers in an R&D division, we could verify the existence of a peak in productivity. For researchers in this case study, it took from 10 to 15 years to achieve outstanding outcomes, after which their productivity reduced. We also found that star researchers had achieved outstanding performance by continuing the same research for a long time. Our results signal a difference between industry and academic researchers. A recent study by Sinatra et al. (2016) suggests that scientists can generate remarkable achievements at any point in their careers. However, researchers in this case study seem to be different. We can argue that these differences are attributable to the way the creative process in both settings, academia, and industry, is subject to differences in freedom of inquiry, motivation, and internal politics. While scientists in Universities and research institutes have some form of autonomy, researchers in industries are expected to contribute to specific topics, constrained by stringent deadlines and budgets. Excellent industry researchers could also have other motivations such as creating their own teams, or trough promotions to climb the even-larger organizational pyramids of Japanese corporations. Finally, industry researchers also need to cope with internal bureaucratic processes imposed by the company and become knowledgeable about the legal and ethical implications not only for their research output but also for the company itself, especially knowledge that takes time to master.

It is worth noticing that contrary to classical studies that propound a proportional link between age and innovation (Lehman, 1953; Simonton's, 1984), the present study deals less with the age of the researcher, but focuses on the time of research engagement, that is, the time researchers have spent in their academic career regardless of their actual age. Although we do not deny the association between the two concepts could be implied. We also point to the fact, as demonstrated in the results, that continued exposure to the same research topics helped to attain remarkable achievements.

By analyzing coinventors, we also observed how star researchers collaborated with others in the organization. When focusing on the subset of star researchers in an R&D division, we did not observe significant spillover effects from stars to non-star researchers. However, we cannot deny interactions among the star researchers and their influence (Grigoriou and Rothaermel, 2014). The expertise should not only be consistent across time but topically shared between inventors. For instance, the R&D division under study could allocate outcome topics into three larger umbrella topics or layers: research on processors, memory, or devices. It seems that focusing on a single technology layer is important, but knowledge also flows among them.

**Figure 5:**
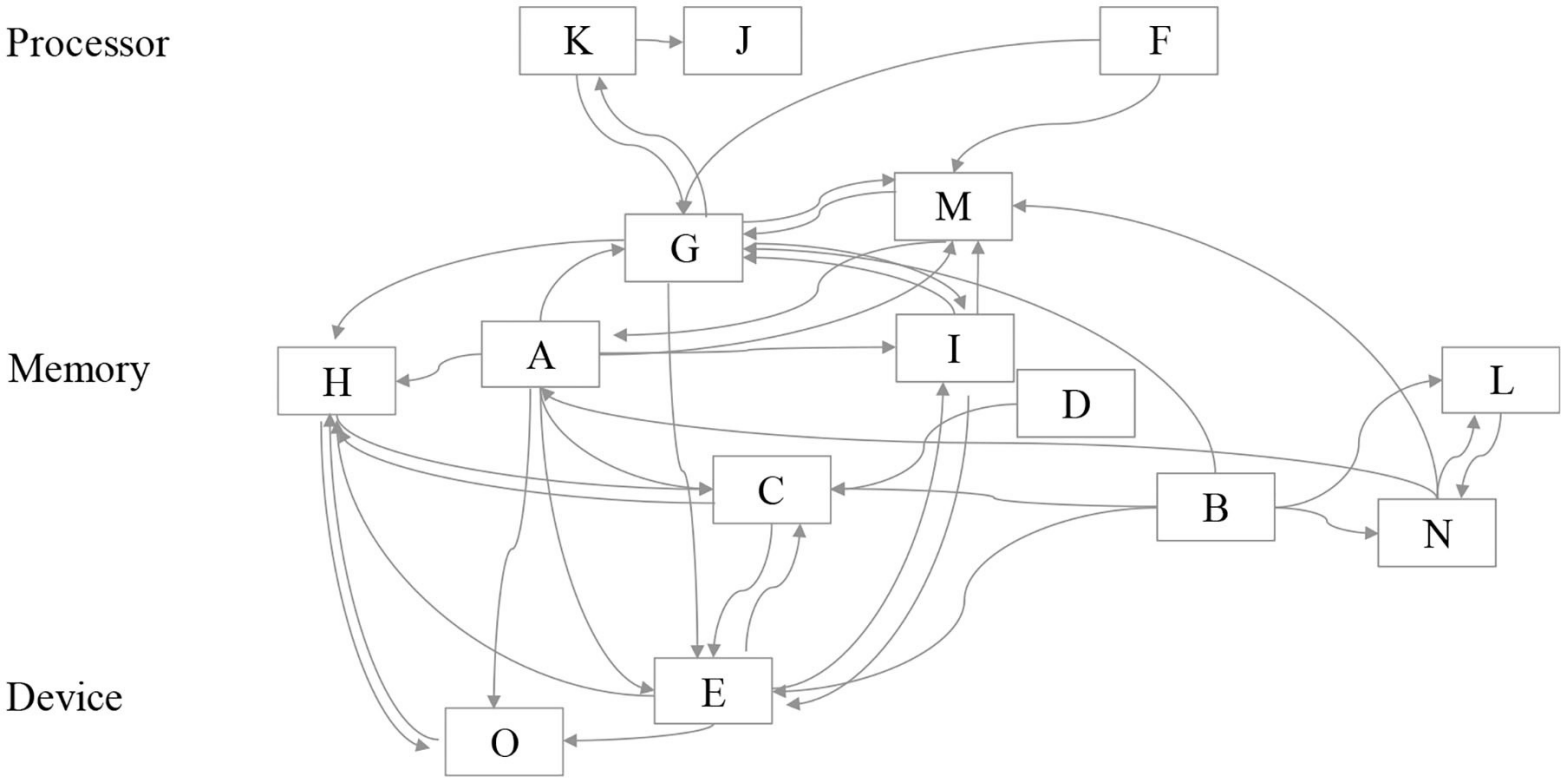
Co-inventor network of star researchers. Each star researcher is placed in the technology layer more closely related to their achievements.

Figure 5 shows the relationship among star researchers in the R&D division. The data are based on patent submissions of stars and co-inventors. The rear end of the arrow represents the primary inventor, whereas the arrowhead represents the co-inventors of these patents. In Figure 5, we can observe the link between star researchers. Based on the classification of patents as primary inventors, we plotted each researcher at different technological layers of the processor, memory, and device. We observe that co-invention happens more frequently across the same layers. For example, researchers -C and -D, who belonged to the same laboratory at the University, are in the same layer, namely memory, and co-invented the patent. A highly-cited patent submitted by researcher-L early in his career was co-invented with researcher-B. Similarly, a highly-cited patent submitted by researcher-O early in his career was co-invented with researcher-E.

Researchers in the R&D division had engaged in developing semiconductors in the 1980s and 1990s when the device size followed Moore's law (Moore, 1998). During that time, researchers were expected to understand the issues to be resolved in each generation of the device, deliberate about how to overcome them by referring to the previous generations, ideate, and demonstrate the feasibility of the ideas by making samples. At each stage, competitors were declaring new ideas or releasing products in the market. If researchers offer indispensable ideas for each shrink-related issue, the company could maintain its competitiveness through patents and related products. Designing the device structure is critical for device fabrication, performance, and circuit. For example, dynamic random-access memory (DRAM) is a generation-type development, and the density of the memory capacity increases gradually by leveraging the shrinkage of the device size. For this type of innovation, it may be an appropriate strategy to focus on R&D in the same layer.

In Figure 5, we can also find linkages among different layers. For instance, researcher-A has multiple interactions with other stars, indicating that he helped other stars. The collaborations included several types of contributions as mentor, manager, or adviser for the patents, although this cannot be observed from the linkage itself. According to the co-invention patent analysis, researchers who had achieved outstanding outcomes at an early stage in their careers were almost always affected by other star researchers. This provides further evidence on the contribution of a diversified technology source of prior art in generating innovative outputs (Battke et al., 2016). Although this has been observed previously with patent citations, in this study we observe it through collaboration ties.

Even during incremental innovations in the semiconductor industry during 1980s and 1990s, communication among different technological layers was indispensable. Process researchers needed to discover ways to fabricate smaller devices through discussions with equipment manufacturers. Device researchers collaborated with process researchers to check the feasibility of the implementation of the device structure and passive components. Circuit designers worked on low voltage operations with a smaller die size to produce competitive products. Critical issues like the hot-carrier, short channel effect, and even quantum effects could arise for device operations when miniaturizing the device. Our results also suggest the importance of interactions among stars, while there is no apparent spillover effect between stars and non-star researchers. This is in line with research suggesting that large in-house capabilities compensate for the lack of spillover from the outside (Grillitsch and Nilsson, 2015), while small and medium size firms benefit from external spillovers based on geographical proximity (Aldieri and Vinci, 2017). In this case, the company is self-sufficient in providing a collaboration network of skilled researchers.

Finally, we tackle performance during the later stage of career development. After a research engagement of more than 15 years, star researchers' productivity decreased. Among the possible causes, we discussed that excellent researchers moved to managerial positions at a later stage of their career. The fact is that, in this study, there are a smaller number of highly-cited patents submitted by researchers at the later stage of their career. However, the issue is about measuring the creative efforts of managers. We cannot declare that there is a decline in the individual's intrinsic creativity, but we still lack the means to properly measure the creative outputs of managers. Moreover, patent activity may only be a part of the story.

Another reason for the decline is due to changes in the market. If a company's efforts to position its proprietary technology fails, then it should move to a new technology. Therefore, if researchers lose in their race for development, not only would they lose their ideas, but expertise also becomes less valuable, and many employees would lose their jobs. In the present case study, the company was engaged in developing CMOS technologies, which were competing against technologies such as NMOS or III-V compound semiconductors, while the other technologies would have captured the market, the interpretation in this study may have been very different.

## Conclusion

In this study, we analyzed the characteristics of star researchers in a Japanese semiconductor company. We analyzed a collaborative network of 6,057 inventors who coinvented multiple of the 44,636 semiconductor-related patents granted in Japan. A group of selected 136 inventors was identified for their remarkable results in terms of patent productivity and impact. A comparison of their collaboration patterns during their career with other inventors with modest contributions helped to uncover the key features of those star researchers. High exposition and collaboration with other stars researchers are the most relevant characteristics that define their remarkable careers.

Additionally, we focused on a selected subset of 15 star researchers based on the recognition of the expert community through awards obtained and belonging to the same R&D division in the company. Analysis of the star researchers' patenting activity shows that outstanding outcomes were obtained after continued engagement on the same or similar topic for 10–15 years. This moment of high production of patents is preceded and followed by periods of comparatively modest productivity. In other words, a hot streak of productivity exists in the context of new product development. Also, we analyzed the knowledge spillover effect of star researchers on co-inventors. Due to a narrow view from the perspective of a single R&D division, the spillover effects could not be directly observed. However, a complete picture of the collaboration ties across the company shows the positive influence of life-long exposition and collaboration with star researchers.

The factors necessary for developing star researchers remain uncertain. Interviews conducted with highly productive researchers or their supervisors tend to point to mentoring by talented professors during their early career stage, the company's market competitiveness, an atmosphere or a sense of crisis and spirit to be the best, and a stimulating research environment.

The phenomenon of hot streaks has also been found in the careers of artists and academics (Liu et al., 2018). The present article shows that it also exists in industries. However, the key difference between productive professionals in other areas is the fact that industry hot streaks, at least for inventors in this case study, tend to appear during a fixed time, after about 10 years of work and not randomly. Moreover, this peak of achievement depends on accumulating expertise in the same area. Hence, changing career or research direction in the same company may be detrimental to a researcher's achievements. Companies must develop strategies to provide their researchers with a stable environment in the long term.

As evident from this study, developing young researchers and passing on the knowledge and experience of interactions among star researchers is the key to a sustainable research process and industrial outcomes. Thus, in addition to talent, an environment that stimulates interaction among the members, regardless of the stages of their career development, is necessary.

The present study aimed to explain the relationship between high-impact productivity and the duration of a researcher's engagement, and several factors that are perceived to be relevant when becoming a star researcher. However, the results presented are limited in scope and aim to outline avenues for future research. For instance, this paper is a specific case study for a prolific Japanese semiconductor company and, more specifically, for researchers in one of its R&D divisions. Other companies in different industries and regions may have their own path-dependent strategies; thus, several other parameters must be studied to reach generalizable results. Our data rely on inventors' collaborative patterns. While patent productivity is staple data for related studies, the impact of patents may extend beyond patent citations. Filing patents in other countries, patent litigation, or patent-licensing are other indicators of impact worth exploring.

## Data Availability Statement

Publicly available datasets were analyzed in this study. This data can be found here: data accessible through https://www.derwentinnovation.com/ by applying the query mentioned in the method section of the article. An anonymized dataset for the replication of the binomial regression model along with an R script is available upon request.

## Author Contributions

AM designed the project, performed the literature search, and conducted the analysis of productivity peak and interviews. CM performed the literature search and conducted the overall assessment of the collaboration network. YK supervised and mentored the project. All authors have made a substantial, direct and intellectual contribution to the study.

## Conflict of Interest

AM was employed by the company Hitachi America, Ltd. The remaining authors declare that the research was conducted in the absence of any commercial or financial relationships that could be construed as a potential conflict of interest.

## Footnotes

^1^The company, star researchers, and executives have opted to remain anonymous.

^2^http://www.mst.or.jp/Portals/0/prize/english/index_en.html

^3^https://www.ieee.org/membership/fellows/index.html
