# Supplementary material for: Collaborative Patterns, Productivity, and Research Impact in the Careers of Star Researchers in a Japanese Semiconductor Company
Source: Front Res Metr Anal. 2020 Sep 29;5:575862. doi: 10.3389/frma.2020.575862 (PMC8028408; doi:10.3389/frma.2020.575862)
Supplement: Supplementary file 1 [file Data_Sheet_1.docx]

**APPENDIX.**

***Summary of interviews.***

To supplement the patent analysis presented in the manuscript, we conducted five interviews, where two of the five interviewees are star researchers, and the rest are department managers from the same company. The reason to include department managers is that in Japanese research laboratories, department managers, who used to work as researchers, lead and provide directions to researchers. They discuss with the star researchers about goal setting and how they can achieve their goals. We interviewed managers using the same questions as those shared with the star researchers. Note that all of them were specialists in semiconductor technologies.

The interviews helped reveal the perceptions of star researchers and others involved in the creative process. The interview contained the following predetermined questions:

What is the difference between start researchers and other researchers? Regarding factors affecting the outstanding outcomes if the researchers are highly productive.

What enables star researchers to achieve outstanding outcomes? Regarding the productivity peak.

Is it reasonable to attain the peak?

How many years does it take to achieve outstanding outcomes?

Why researchers cannot achieve outstanding outcomes in the early or late stage of their research career?

**Interview to researcher -H**

| **Characteristics of star researchers** | A researcher who generates ideas and executes them. Carries a spirit of perseverance and adapts flexibility in the research process. |
| --- | --- |
| **Factors affecting the outstanding outcomes** | During the 1980s and 1990s, the semiconductor memory business was very promising, and many companies were competing against each other. My company focused on the business and decided to make a substantial effort toward R&D, which would then determine its future. The research laboratory had an environment of demonstration right from wafer, design, trial, to proof of device in a single stretch. The target of our research, DRAM, was clear, and the level of difficulty was appropriate as the development was incremental and a generation-type advancement. I personally did not want to follow incremental ideas but suggested not-too-aggressive but proper ideas. We also had a great mentor in star researcher-A. This type of environment was key to our achievements. |
| **Comment on the peak of creative accomplishments** | During the early stages of their research careers, researchers executed jobs that their supervisors expected them to complete. After engaging with research for nearly ten years, they would understand what was expected of them and could see the overview and meaning of their research from both technological and business perspectives. The decline in the outcome is not about creativity but about job transition from a researcher to a leader of the research group. However, when I recall now, I am not sure if I really devoted myself to the theme in my later career as a researcher. |

**Interview to researcher -O**

| **Characteristics of star researchers** | A researcher who continues to think. In a negative sense, you can call that stubbornness but the ability to keep thinking is very important. You can imagine the attitude of Shuji Nakamura, who received the Nobel Prize in physics, for developing blue LED by continuing to research his own. The key to continuing the research was not doggedness, but adopting a smooth approach under the company’s code. |
| --- | --- |
| **Factors affecting the outstanding outcomes** | We experienced an atmosphere of competition, as we aimed to become number one in the world. Moreover, we also understood the dangers of development and market competitiveness. During my second year in the company, I came up with an idea. I read fundamental papers related to the theme and tried to understand the principle. Another perspective was that I was instructed to understand deeply as well as get an overview of the research both during my graduate years at the university and the company itself. As for the idea itself, there was a similar structure in a paper, but I considered a structure that was technologically feasible. |
| **Comment on the peak of creative accomplishments** | I think ideas were generated earlier than the research engagement of ten years, but it just took years to reveal or come into the public domain. I believe young researchers had creative ideas compared to senior investigators. |

**Interview to department managers**

| **Characteristics of star researchers** | It is important to have different perspectives on what a typical researcher from the domain possesses. For that, holding discussions with other researchers is an important aspect. (Principal researcher A) |
| --- | --- |
|  | It is necessary to have a deep understanding of the technology as well as to perceive things from a wider perspective. Also, star researchers tend to transmit information in and out of the company. (Project Manager B) |
|  | It depends on their character, but I am not sure what it is. (Department Manager C) |
| **Comment on the peak of creative accomplishments** | It may take ten years or more to understand the technology and approach for research including the in-house processes. (Project Manager B) |
|  | The result of peaking in creativity and a few major achievements at an early and later stage of research career fits into my sense. When a researcher grows older, it is not easy to suggest ideas. However, if a person can, I do not know the difference between who can and cannot.  (Department Manager C) |

From the interviews, we observed that star researchers acknowledged the value of collaboration. One of the star researchers was able to produce creative ideas that resulted in more than ten patents during the early stages of their career (i.e., researcher -O). The creative process was not simple and involved close help from others. Two other interviewees reported that they were significantly influenced by their mentors.

From the interviews, we observed the concerns regarding the competitive environment where multiple technologies grapple to capture the largest market share.

The two star researchers reported that it is possible to generate impactful ideas at a later stage of their career, also reporting that the energy put into each project as a direct creator was less than during their earlier years.
